# Effect of UV-Absorbing Nets on the Performance of the Aphid Predator *Sphaerophoria rueppellii* (Diptera: Syrphidae)

**DOI:** 10.3390/insects11030166

**Published:** 2020-03-05

**Authors:** Rocco Amorós-Jiménez, María Plaza, Marta Montserrat, M. Ángeles Marcos-García, Alberto Fereres

**Affiliations:** 1 BioNostrum Pest Control S.L., C/Martillo 40A San Vicente del Raspeig, 03690 Alicante, Spain; 2 Instituto de Ciencias Agrarias (ICA-CSIC), C/Serrano 115 dpdo., 28006 Madrid, Spain; 3 Instituto de Hortofruticultura Subtropical y Mediterránea “La Mayora” (IHSM-UMA-CSIC), Avenida Dr. Wienberg, s/n, Algarrobo-Costa, 29750 Malaga, Spain; 4 Centro Iberoamericano de la Biodiversidad (CIBIO-UA), Carretera San Vicente del Raspeig s/n, San Vicente del Raspeig, 03690 Alicante, Spain

**Keywords:** Syrphidae, UV-absorbing net, fitness, density, dispersion, foraging behavior

## Abstract

Photoselective nets have proven to be effective for aphid pest control as they limit their dispersal ability. However, little is known on the impact of such nets on natural enemies of aphids. In this work, we study the effect of UV-absorbing nets on the syrphid fly *Sphaerophoria rueppellii* Wiedemann (Diptera: Syrphidae), a commercially available aphid biocontrol agent in Mediterranean horticultural crops. First, we released mature syrphid adults and evaluated density and dispersal of the resulting immatures in a turnip crop grown under either UV-blocking (Bionet) or standard net. Second, we assessed, under controlled conditions, the impact of UV radiation on fitness-related parameters, and on flight behavior of *S. rueppellii* adults. Results showed that, while syprhid immature density was higher, their dispersion was reduced under Bionet. UV-absorbing nets are known to influence the dispersion pattern of aphids, which may have indirectly conditioned the distribution of their predator *S. rueppellii*. On the other hand, the type of net had no influence on the performance of adults. We conclude that the use of photoselective nets and the release of syrphid predators such *S. rueppellii* are compatible strategies to be used in IPM aphid-control programs.

## 1. Introduction

Aphids (Hemiptera: Aphididae), particularly species such as *Aphis gossypii* Glover, *Macrosiphum euphorbiae* (Thomas), or *Myzus persicae* Sulzer, are among the most destructive and abundant pests of horticultural crops worldwide [1]. The damage aphids cause through feeding, the large amount of honeydew they produce, and their efficiency as vectors of plant viruses, make them one of the most damaging pests in protected crops [2,3,4].

In recent years, development of pesticide resistance by aphids and legal restrictions to pesticide use have stimulated the implementation of Integrated Pest Management (IPM) strategies for aphid pest control, in both indoor and outdoor crops [5,6]. One of such IPM strategies is the use of UV-absorbing covers that block part of the UV radiation transmitted inside greenhouses. UV-absorbing covers have proven to be effective at reducing the incidence of pests and diseases in commercial greenhouses [7,8]. Alteration of the UV part of the solar spectrum perceived by insects affects behavioral traits such as spatial orientation, navigation, feeding, and mating behavior [9,10]. Recent studies showed that UV-deficient environments reduce winged aphids flight and reproduction ability, thereby reducing aphid propagation and dispersal rate within greenhouses [5,11,12]. The success of IPM strategies involving the use of photoselective covers for aphid control requires not only the understanding of the effects of these covers on the insect pest and their host plants, but also on their natural enemies [13]. Surprisingly, only few works have studied the effects of altering UV lightning on natural enemies of aphids, most of them focused on parasitoids [5,14] and very few on predators [15]. Therefore, at present, more research on the effects of these materials on aphid predators is needed [8].

This work aims at evaluating the effect of UV-blocking nets on the spatiotemporal dynamics of an aphid predator, the syrphid fly *Sphaerophoria rueppellii* Wiedemann (Diptera: Syrphidae). This species is the most abundant predatory syrphid in Mediterranean greenhouse crops, and it is already a commercially available biocontrol agent for aphid control in greenhouses [16,17]. The experimental system involved turnip [*Brassica napus* L. (Cruciferae)] and sweet pepper [*Capsicum anuum* L. (Solanaceae)] plants infested with the aphid *M. persicae*, a species that causes severe economic losses in numerous crops worldwide [18,19]. Field experiments and bioassays under controlled conditions were carried out to explore the effects of UV-blocking covers on dispersal, foraging behavior, and fitness-related parameters of the syrphid.

## 2. Materials and Methods

### 2.1. Plant Material, Aphids and Syrphids

Sweet-pepper [*C. annuum* L. (Solanaceae) var. California Wonder], turnip (*B. napus* var. Just Right), and sweet alyssum [*Lobularia maritima* L. (Brassicaceae)] plants were grown in a climate room (T = 22 °C ± 2 °C, RH = 80% ± 10%, Photoperiod = 16L:8D) in plastic pots (5 × 5 × 6 cm). Sweet-pepper plants were used to maintain a stock colony of *M. persicae* in mesh netting cages (50 × 35 × 35 cm) in a climate room at the same conditions described above. *Sphaerophoria rueppellii* adults were also reared in mesh netting cages (60 × 60 × 80 cm) containing bee pollen from various flowers, sucrose, water, and a sweet-pepper plant infested with *M. persicae* to stimulate oviposition. Same-aged syrphid eggs were periodically removed from the adult cultures and the emerged larvae were reared until pupation on sweet-pepper plants infested with *M. persicae*.

### 2.2. Field Experiments

#### 2.2.1. Experimental Design

Experiments were conducted in “La Poveda”-CSIC, an experimental farm located in Arganda del Rey, Madrid, Spain (40°18′ N, 3°26′ W). Two “tunnel type” nethouses (6.5 m wide, 8 m long and 2.6 m height) with the same orientation (N-S) and separated five meters from each other were used to compare two types of nets: A standard 50 mesh net (Criado y López, El Ejido, Spain) with no special UV-absorbing properties, and Bionet (Meteor Agricultural Nets, Ltd., Petach-Tickva, Israel) which filters about 40% radiation in the UV spectrum. These two nets have similar physical properties (mesh size, open area, mechanical strength). The two nethouses contained four compartments (6.5 m wide, 2 m long, and 2.6 m high), each one isolated from the adjacent with walls made of standard 50 mesh net. Compartments constitute the experimental units of the experiment. Because alternating the two types of nets in the compartments within each nethouse could cause undesirable shading from adjacent covers with photoselective properties of the other treatment, all four compartments of each nethouse were covered with the same type of net. Nested models were applied to account for potential effects derived from such experimental design (see ‘Statistical analysis’).

To corroborate that UV-blocking differed between the two types of nets, the UV radiation transmitted inside each compartment was measured before every insect count using a portable quantum meter radiometer sensitive to ultraviolet radiation (320–400 nm) (Apogee, Logan, UT, USA). Temperature and relative humidity inside each compartment were registered using data loggers (Tinytag Ultra 2s, Gemini Data Loggers, Chichester, UK). These measures were taken because climate conditions inside greenhouses are a key factor influencing the performance of natural enemies, since it determines establishment and reproduction of adults, as well as development and survival of immature stages [20,21].

In each compartment, 66 turnip plants at the 2-leaf stage were transplanted in early September 2010, distributed in 11 rows. Plants were spaced 0.3 m along water drip rows, and 0.5 m between rows. On the same day, five flowering sweet alyssum plants were transplanted in the corners and the center of each compartment, to serve as food source for syrphid adults. This plant species is very attractive for hoverflies, and several works have demonstrated its suitability as insectary plant [22,23,24]. One week after transplanting, a Petri dish containing 80–100 *M. persicae* winged adults was placed in the center of each compartment to promote a natural infestation into the crop. An initial density of 5–10 aphids per plant in each compartment was achieved a week later. At this moment, three mature 7 day-old females and two males of *S. rueppellii* were released in each compartment. These syrphids came from a mass-reared colony originally started at the CIBIO research institute (Alicante, Spain), and maintained as described above.

#### 2.2.2. Aphid and Syrphid Samplings

Monitoring of aphid and syrphid population was conducted twice a week from 24 September to 8 November 2010. At the beginning of samplings, turnip plants were at the growth stage 16, according to the extended BBCH scale [25]. The number of aphids and syrphid immature stages (eggs and larvae) were counted by thorough inspection of 7 turnip plants randomly selected. Aphid abundance on each plant was grouped into five categories depending on population density, following the same scale as described in [11]: 0: 0 aphids; 1: 1–4 aphids; 2: 5–19 aphids; 3: 20–49 aphids; 4: 50–149 aphids; 5: ≥150 aphids. Aphid stages (nymphs, apter, or alate adults) were not counted separately because aphid density rather than stage structure of the aphid colony is the main factor influencing egg production and oviposition in *S. rueppellii* females. Indeed, *S. rueppellii*’s larvae successfully develop into adults when they are left to feed on all aphid stages (Amorós-Jiménez & Marcos-García, unpublished results).

### 2.3. Effects of UV on Fitness-Related Parameters and Foraging Behavior

The experiments were carried out in insect-proof cages allocated inside a glasshouse under controlled conditions (25 °C ± 2 °C, RH = 60% ± 10%, Photoperiod = 12L:12D), at the ICA-CSIC (Madrid, Spain). Cages were covered with either photoselective or standard nets. Because light naturally entering the glasshouse strongly depended on the local weather, UV values registered inside the two types of cages often did not differ. To better mimic radiation during sunny days, three special lamps (ULTRA-VITALUX, Osram, Spain) producing light similar in its composition to the mixture of natural sunlight were placed above the cages as additional light sources. As a result, the percentage of UV radiation transmitted inside standard-netting cages was on average 38% higher than that measured under the photoselective nets, in all the experiments (Table S1). Cages were placed on top of a table (5 m long × 1.81 m wide) oriented N-S. Relative position of cages, and orientation on the table, was randomized on a daily basis to account for possible differences in light intensity caused by the structure of the glasshouse, or by shadows from other structures outside the glasshouse. UV radiation was measured using the same device described above.

#### 2.3.1. Fitness-Related Parameters

To assess the effect of photoselective covers on *S. rueppellii*’s fecundity and fertility, one newly emerged female and two males were placed in a cage (40 × 40 × 40 cm) covered by either Bionet or standard net. Preliminary studies revealed that the size of the cages is suitable to perform flight and choice experiments (Amorós-Jiménez et al. unpublished results). Each cage contained food, water, and a sweet-pepper plant infested with approximately 300 individuals of *M. persicae* at the start of the trials. Syrphids were left seven days in these conditions to ensure mating. After this period, each plant was observed by visual inspection to confirm that oviposition had taken place, and females that had not laid eggs were discarded. Each mature female was transferred to a cage covered with standard net that contained a new aphid-infested plant. After 48 h, the eggs laid on this plant were counted to evaluate syrphid fecundity (i.e., number of eggs in 48h). To quantify fertility (i.e., fraction of eggs hatching), leaves containing eggs were cut off from the plant, and placed in a Petri dish (90 × 15 mm) with a wet disk of paper inside, and sealed with Parafilm^®^ to achieve the high relative humidity conditions necessary for egg development to complete [16]. Ten replicates were performed for each treatment.

#### 2.3.2. Foraging Behavior

To test the effect of UV-blocking materials on the recognition of floral resources by adults, we used mesh-netting cages (100 cm long, 60 cm wide, and 60 cm high) covered with either the standard 50 or the Optinet 50 mesh (Polysack Plastic Industries, Ltd., Nir Yitzhak, Israel), the latest with similar UV-absorbing properties than the Bionet 50 mesh. A flowering sweet alyssum plant was placed in one of the extremes of the cage. A 2–4-day-old syrphid female was placed in the opposite side of the cage, inside of a glass tube placed on a flying platform 30 cm above the ground. The tube was covered with black adhesive tape to help syrphids to orientate towards the tube opening. All females were naïve, in the sense that they never experienced the test conditions before, and each female was used only once. The time devoted to recognize the floral resource (i.e., time until the syrphid performs either a suspended flight near the plant or flies in circles around it) was recorded using the behavioral observation program Etholog [26]. Observations lasted a maximum of 15 min. Females that did not exhibit this behavior during the interval were also included in the statistical analyses (see below). Ten replicates were performed for each cover.

### 2.4. Statistical Analysis

UV radiation, temperature, and relative humidity under each type of net were analyzed separately with Generalized Linear Models (GLM), which included two main factors: ‘nethouse’ and ‘compartment’ nested to ‘nethouse”. We assumed a Normal error structure of data and a logit relationship between the environmental data and the lineal combination of the explanatory variables.

The abundance of aphids (scale value) and syrphid immature stages on each type of nethouse were analyzed using Generalized Linear Mixed Models (GLMM), which allow for non-linearity, non-constant variance, and clustered structure of data, including time as random variable, the main factor ‘compartment’ nested to ‘nethouse”, and assuming a Poisson error structure of data and a logit relationship between the response variable and the explanatory variables [27,28]. Additionally, data on presence–absence of syrphid immatures on turnip plants were analyzed with GLMM adjusted to a Binomial distribution with logit link function, as proxy for the dispersion of immature stages within each type of net.

Fecundity and fertility data followed a normal distribution and were analyzed with t-tests. Fertility data were log-transformed to achieve homoscedasticity. To detect whether nets interfered differentially with the foraging behavior of syrphids, the time needed by each individual to recognize flowers was compared between type of nets using Kaplan–Meier estimators followed by log-rank test for pairwise comparisons [29]. This analysis permits the inclusion of replicates in which the event (flower recognition) does not occur (i.e., censored data [29]. All data were analyzed with the statistical package SPSS V20.0. (IBM Co., NY, USA).

## 3. Results

### 3.1. Field Experiments

#### 3.1.1. Light Properties and Environmental Conditions

UV radiation was significantly higher under the standard net than the Bionet (22.82 ± 0.78 and 10.61 ± 0.35 μmol/m^2^ s, respectively: χ^2^ = 220.97, df = 1, *p* < 0.001). There were no differences in UV radiation among compartments within each nethouse (χ^2^ = 8.92, df = 6, *p* = 0.18).

Mean daily temperature was significantly lower under the photoselective than under the standard cover (15.77 ± 0.31 and 17.36 ± 0.31 °C, respectively: χ^2^ = 4.94, df = 1, *p* = 0.03), a result that agrees with previous works [11,30], whilst there were not statistical differences among compartments within each nethouse (χ^2^ = 3.01, df = 4, *p* = 0.56). Despite the fact that the average temperature under the two nets was below the theoretical range for optimal development and performance of *S. rueppellii* [16], both adults and immatures seemed to be well adapted to the nethouse conditions. Indeed, this species is able to locate and exploit microhabitats within plants that provide shelter and optimal abiotic conditions [16]. 

The relative humidity was not different between the two types of net (68.29 ± 0.86 and 69.73 ± 1.05%, respectively: χ^2^ = 1.12, df = 1, *p* = 0.29), neither among compartments within nethouses (χ^2^ = 0.49, df = 3, *p* = 0.92). Average relative humidity of all compartments was always above the critical value for *S. rueppellii* immature development (i.e., 60%) [16].

#### 3.1.2. Light Properties and Environmental Conditions

**Figure 1:**
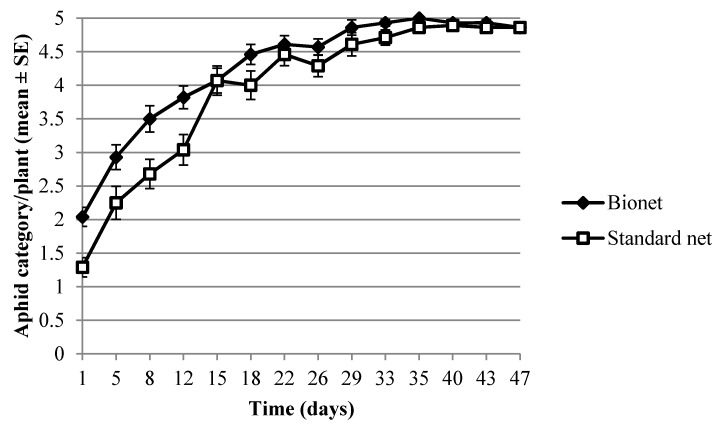
Mean ± SE of the temporal evolution of aphids (scale value), in the nethouses covered with Bionet and Standard net.

The average density of aphids was significantly higher under the photoselective than under the standard net throughout the experiment (4.18 ± 0.05 and 3.78 ± 0.05, respectively) (Table 1). However, this effect was probably caused by aphid densities being higher under the Bionet than under the standard net from the beginning (Figure 1). The analysis also detected differences in aphid density among compartments within nethouses (Table 1).

#### 3.1.3. Density of Syrphid Eggs

**Figure 2:**
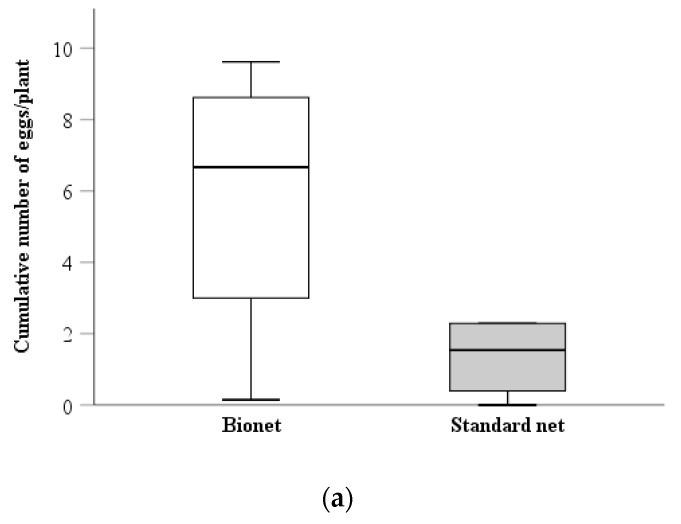

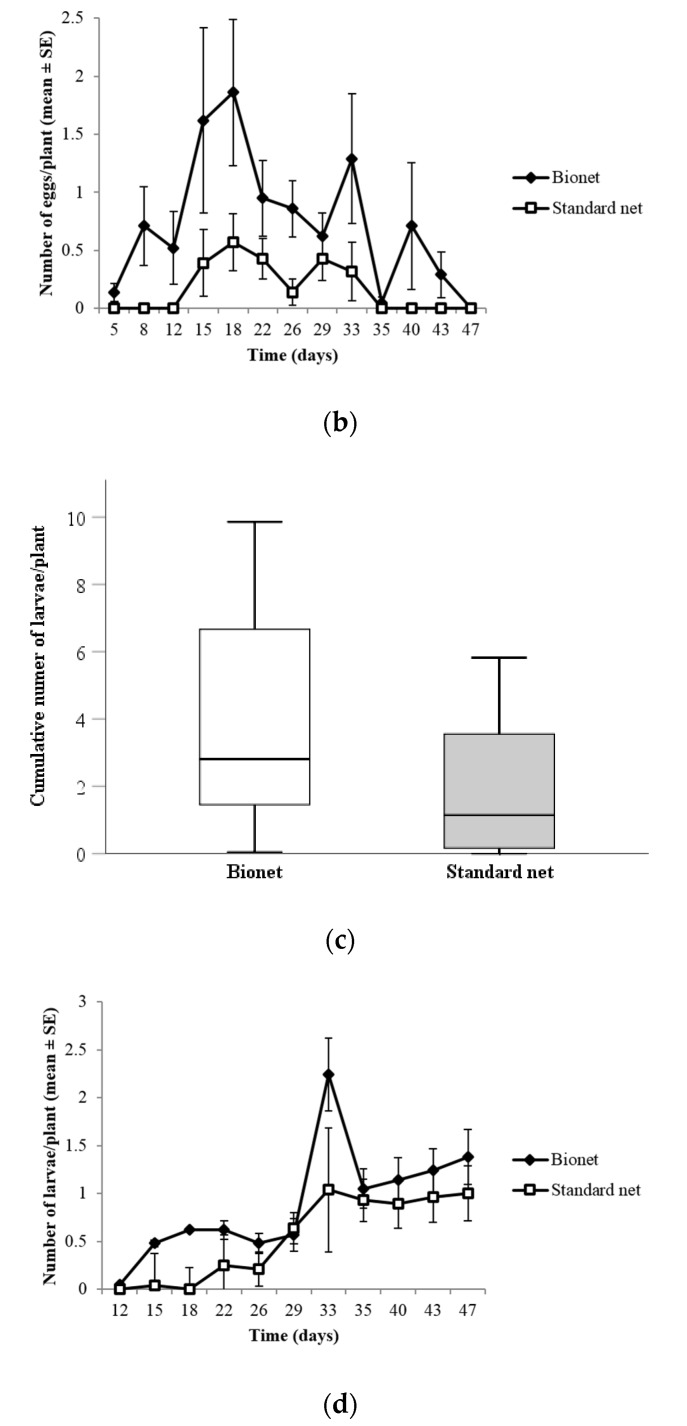
Cumulative abundance and temporal evolution (Mean ± SE) of syrphid immature stages under each type of net: (**a**) Cumulative number of syrphid eggs; (**b**) syrphid egg counts; (**c**) cumulative number of syrphid larvae; (**d**) syrphid larvae counts.

The average number of syrphid eggs per plant was remarkably higher under the photoselective than under the standard cover (0.61 ± 0.09 and 0.18 ± 0.04, respectively) (Table 1, Figure 2a), whereas no other significant sources of variation were detected. Figure 2b shows that syrphid egg-laying started a week earlier, and it lasted longer, under the Bionet than under the standard cover. None of the sources of variation from the analysis of the spatial distribution (presence/absence) of syrphid eggs were significant; consequently, no conclusions could be made on dispersal of females based on egg distribution.

#### 3.1.4. Density of Syrphid Larvae

The abundance of *S. rueppellii*’s larvae was also markedly higher under the photoselective than the standard net (0.71 ± 0.08 and 0.31 ± 0.05, respectively) (Table 1, Figure 2c). Larvae were observed earlier, and at higher densities, under the Bionet than on the standard net (Figure 2d). Interestingly, the analysis of the spatial distribution of syrphid larvae revealed that under the standard net, a higher number of plants had presence of larvae, relative to the UV-blocking net (Table 1), suggesting that dispersal of larval stages was higher under standard netting.

### 3.2. Effects of UV on Fitness-Related Parameters and Foraging Behavior

#### 3.2.1. Fitness-Related Parameters

The number of eggs laid by mature females after 48 h was not significantly different between the two treatments (75.55 ± 13.56 standard and 60.5 ± 14.38 Bionet: t19 = 0.76, *p* = 0.46). Similarly, no significant differences in fertility were found (58.09 ± 6.58 standard and 44.5 ± 4.17 Bionet: t19 = 1.61, *p*= 0.12).

#### 3.2.2. Foraging Behavior

**Figure 3:**
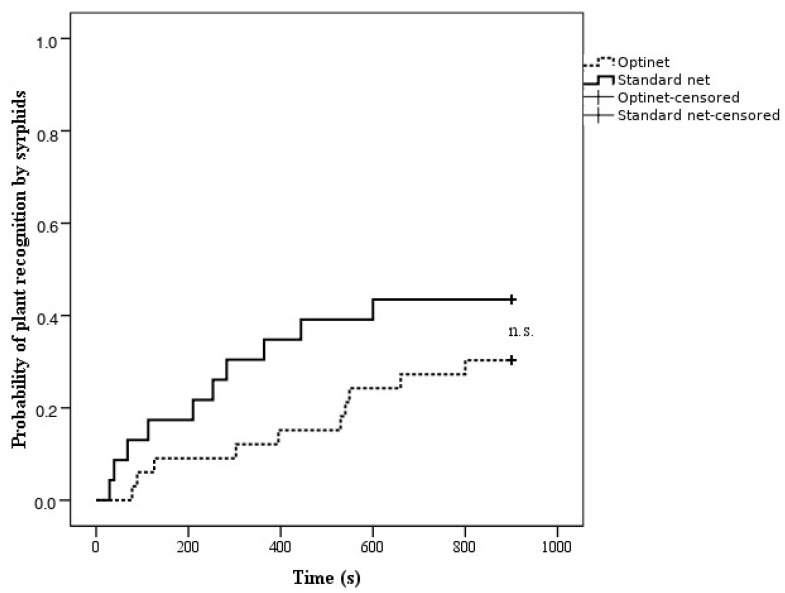
Cumulative proportion of syrphid females recognizing the flowering plant (sweet alyssum) (Kaplan–Meier) under Optinet (n = 33, dotted line) and Standard net (n = 23, drawn line).

The proportion of syrphids that displayed flower-recognition behavior did not differ between nets. The probability of plant recognition by syrphid adults along time did not differ between treatments (χ^2^ = 1.54, df = 1, *p*= 0.21) (Figure 3).

## 4. Discussion

Field and lab experiments were carried out to determine whether UV radiation influenced the performance of the aphidophagous syrphid *S. rueppellii*. In our experiments, the density of both egg and larval stages of *S. rueppellii* was higher under the Bionet mesh, suggesting that photoselective covers may enhance fecundity or fertility of the released females. Yet, life-history experiments indicated that these traits were not influenced by the lack of UV radiation. Density-dependent oviposition may provide an alternative and more likely explanation. In syrphids, oviposition site choice is known to depend on multiple factors, such as host plant characteristics, aphid species, presence of other predators, and prey abundance [31]. Several studies have shown that syrphids adjust the number of eggs laid to the size of the aphid colonies, to maximize the survival probability of their offspring [32,33,34]. In fact, aphid depletion before larvae complete their development might result in death by starvation and/or cannibalism [31]. The higher number of immature stages found under the Bionet mesh may have been caused by female syrphids adjusting their oviposition rate to the denser aphid colonies found under these nets. Indeed, aphid density was consistently higher under the photoselective than the standard net, although probably due to an initial higher density of aphids under Bionet. This higher density would also imply higher presence of volatiles and honeydew produced by the aphid colonies, which are known to stimulate oviposition in syrphids [35,36]. Furthermore, syrphid eggs were not observed until aphid density reached the same category of infestation under both nets (20–49 aphids per plant), which may indicate that at lower aphid densities the stimuli provided by the prey do not surpass the acceptance threshold of *S. rueppellii* females [37].

Our results also suggested that syrphid larvae dispersed more under the standard net. Syrphid larvae are able to move between plants in search for new aphid colonies [38], and dispersion is often triggered when aphid availability within a plant is insufficient to support larval development to maturity [39,40]. In addition, it is known that UV-blocking nets have a negative effect on dispersal and propagation of aphids, through a lower production of alate forms and a reduced motor activity [5,12], which leads to higher aphid aggregation patterns in crops under UV selective nets [41]. We hypothesized that because the standard netting does not interfere with aphids’ dispersal, syrphid larvae probably found smaller aphid colonies under the standard nets, which in turn triggered their moving between plants in search for new food patches. Indeed, *S. rueppellii* larvae are able to survive under low aphid availability conditions, by exploiting small and developing aphid colonies in different plants [16].

Syrphid females require proteins and amino acids from pollen for their ovaries to mature and to sustain egg production, and nectar to gain energy to search for oviposition sites [42]. Therefore, factors hampering syrphids to find flowers could easily result on lower syrphid density in the crop. In this work we also evaluated whether UV-blocking nets reduced the ability of syrphid adults to find flowering plants they use as food sources. Our results suggested that this was not the case, as the probability of adult syrphids to find flowers did not differ under the two types of nets. However, our results are not conclusive onto whether UV-blocking nets affect syrphid visual perception of flowers, as syrphids are still capable of using other senses to locate food sources. Indeed, while optical stimuli are important for syrphid choice-making behavior [43], volatile compounds also play a key role in the attraction of syrphids towards flowering plants [44]. At the greenhouse’s scale, syrphids are in close contact with plants and their herbivores, and such olfactory cues might be more important than visual cues for hoverfly detection of, and orientation towards, their food sources, as well as their oviposition sites.

Knowing the degree of compatibility between biological control agents and the use of UV- absorbing covers is crucial for successful pest management in protected crops [12]. Our results suggest that UV-deficient environments induced under photoselective screens do not have a negative effect on population dynamics or performance of the predator *S. rueppellii*. Instead, UV- radiation indirectly influences the abundance, and shapes the distribution of this predator within the crop, as it affects the dispersal of their prey. As it has been shown in other studies, while natural enemies that mainly rely on their vision may have difficulties on their host/prey searching behavior under UV-deficient environments [12,14], predatory syrphids are able to efficiently find aphid patches attracted by the blend of volatiles produced by plants and by aphid colonies [45]. Our results suggest that they may be able to do so also under low UV radiation environments. 

## 5. Conclusions

Based on our results, we can conclude that the use of UV-absorbing nets and releases of the predatory syrphid *S. rueppellii* are compatible strategies to be incorporated into IPM programs against aphid pests. Our research contributes significantly to the available information regarding the effects of UV-blocking barriers on aphid predators, which remained mostly unknown.

## Figures and Tables

**Table 1:** Resume table of Generalized Linear Mixed Models (GLMM) models. Density and presence–absence data were adjusted to a Poisson and a Binomial model, respectively.

Parameter	Source of Variation	F	df	*p*
Aphid density	Nethouse (1)	36.572	1, 768	<0.001
	Compartment (nethouse) (2)	3.854	6, 768	0.001
	Time (3)	578.189	1, 768	<0.001
	(1) * (3)	19.483	1, 768	<0.001
	(2) * (3)	1.315	6, 768	0.248
Syrphid egg density	Nethouse (1)	4.167	1, 623	0.042
	Compartment (nethouse) (2)	1.414	4, 623	0.228
	Time (3)	2.697	1, 623	0.101
	(1) * (3)	0.001	1, 623	0.976
	(2) * (3)	0.530	4, 623	0.714
Syrphid egg presence-absence	Nethouse (1)	0.000	1, 77	0.999
	Compartment (nethouse) (2)	0.551	5, 77	0.737
	Time (3)	0.000	1, 77	1.000
	(1) * (3)	0.000	1, 77	1.000
	(2) * (3)	0.168	5, 77	0.974
Syrphid larvae density	Nethouse (1)	5.368	1, 525	0.021
	Compartment (nethouse) (2)	2.224	5, 525	0.051
	Time (3)	50.918	1, 525	<0.001
	(1) * (3)	1.539	1, 525	0.215
	(2) * (3)	0.916	5, 525	0.470
Syrphid larvae presence-absence	Nethouse (1)	4.808	1, 63	0.032
	Compartment (nethouse) (2)	0.721	5, 63	0.610
	Time (3)	82.345	1, 63	<0.001
	(1) * (3)	1.396	1, 63	0.242
	(2) * (3)	1.229	5, 63	0.306

## Supplementary Materials

The following are available online at https://www.mdpi.com/2075-4450/11/3/166/s1, Table S1: Percentage of UV radiation transmitted under each type of net (mean ± SE). Different letters between Standard net and Photoselective net refer to significant differences (student t-test, *p* < 0.05).
