# Supplementary material for: Effect of UV-Absorbing Nets on the Performance of the Aphid Predator Sphaerophoria rueppellii (Diptera: Syrphidae)
Source: Insects. 2020 Mar 5;11(3):166. doi: 10.3390/insects11030166 (PMC7142947; doi:10.3390/insects11030166)
Supplement: Supplementary file 1 [file insects-11-00166-s001.pdf]

Table S1. Percentage of UV radiation transmitted under each type of net (mean ± SE). Different letters between Standard net and Photoselective net refer to significant differences (student t-test, P < 0.05).

Experiment	Standard net	Photoselective net
Field experiment	48.93 ± 1.30a	20.39 ± 0.88b
Fitness-related parameters	99.46 ± 7.56a	61,63 ± 5.01b
Foraging behaviour	87.04 ± 6.01a	49.29 ± 4.43b
